# Fish Oil Decreases Hepatic Lipogenic Genes in Rats Fasted and Refed on a High Fructose Diet

**DOI:** 10.3390/nu7031644

**Published:** 2015-03-05

**Authors:** Gabriela S. de Castro, João Felipe R. Cardoso, Philip C. Calder, Alceu A. Jordão, Helio Vannucchi

**Affiliations:** 1 Department of Internal Medicine, Faculty of Medicine of Ribeirão Preto, University of São Paulo, Av. Bandeirantes 3900, Ribeirão Preto, SP, 14049-900, Brazil; E-Mails: alceu@fmrp.usp.br (A.A.J.); hvannucc@fmrp.usp.br (H.V.); 2 Human Development and Health Academic Unit, Faculty of Medicine, University of Southampton, Tremona Road, Southampton, SO16 6YD, UK; E-Mail: pcc@soton.ac.uk; 3 Department of Pathology Faculty of Medicine of Ribeirão Preto, University of São Paulo, Av. Bandeirantes 3900, Ribeirão Preto, SP, 14049-900, Brazil; E-Mail: jfcardoso@usp.br

**Keywords:** fish oil, omega-3, fructose, fasting, refeeding, liver

## Abstract

Fasting and then refeeding on a high-carbohydrate diet increases serum and hepatic triacylglycerol (TAG) concentrations compared to standard diets. Fructose is a lipogenic monosaccharide which stimulates *de novo* fatty acid synthesis. Omega-3 (*n*-3) fatty acids stimulate hepatic β-oxidation, partitioning fatty acids away from TAG synthesis. This study investigated whether dietary *n*-3 fatty acids from fish oil (FO) improve the hepatic lipid metabolic response seen in rats fasted and then refed on a high-fructose diet. During the *post-prandial* (fed) period, rats fed a FO rich diet showed an increase in hepatic peroxisome proliferator-activated receptor α (PPAR-α) gene expression and decreased expression of carbohydrate responsive element binding protein (ChREBP), fatty acid synthase (FAS) and microsomal triglyceride transfer protein (MTTP). Feeding a FO rich diet for 7 days prior to 48 h of fasting resulted in lower hepatic TAG, lower PPAR-α expression and maintenance of hepatic *n*-3 fatty acid content. Refeeding on a high fructose diet promoted an increase in hepatic and serum TAG and in hepatic PPAR-α, ChREBP and MTTP expression. FO did not prevent the increase in serum and hepatic TAG after fructose refeeding, but did decrease hepatic expression of lipogenic genes and increased the *n*-3 fatty acid content of the liver. *n*-3 Fatty acids can modify some components of the hepatic lipid metabolic response to later feeding with a high fructose diet.

## 1. Introduction

Fasting increases the rate of triacylglycerol (TAG) lipolysis in adipose tissue, resulting in release of non-esterified fatty acids (NEFA) into the bloodstream. These are used by various tissues as energy substrates. In the liver NEFA oxidation provides the energy necessary to support gluconeogenesis. Thus, fasting is characterised by TAG mobilisation and fatty acid oxidation. In the *post-prandial* (fed) state, lipid metabolism is dominated by secretion of TAG-rich lipoproteins from the enterocytes into the circulation from where the TAG fatty acids are cleared by adipose tissue for storage, with remnant lipoprotein particles being cleared by the liver. Components of these may be assembled into very-low density lipoproteins which are released from the liver again, delivering TAG to the circulation. Furthermore, in the fed state carbohydrate is utilised by *de novo* hepatic fatty acid synthesis, with the fatty acids formed being incorporated into TAG and being secreted into the bloodstream as components of very-low density lipoproteins. Peroxisome proliferator-activated receptor α (PPAR-α) is a nuclear receptor that controls the hepatic lipid metabolic response to altered physiological states [1]. Hepatic expression of PPAR-α is increased during fasting resulting in higher rates of β-oxidation due to enhanced transcription of genes involved in fatty acid transport and oxidation [2].

Fructose is a highly lipogenic monosaccharide whose consumption in the human diet increased after the 1970s due to industrial production and use of high fructose corn syrup [3]. In animal models a high-fructose diet results in increased hepatic lipogenesis and TAG accumulation, elevated plasma TAG concentrations and insulin resistance [3].

Omega-3 (*n*-3) polyunsaturated fatty acids are known to modulate hepatic gene expression resulting in increased β-oxidation and decreased lipogenesis, effects mediated through PPAR-α and its target genes [4]. Consequently *n*-3 fatty acids are able to decrease plasma TAG concentrations [5,6] with subsequent improvements in cardiovascular morbidity and mortality, and in other inflammatory conditions [7,8]. Fish oil (FO) is a rich source of the main bioactive *n*-3 fatty acids, eicosapentaenoic acid (EPA) and docosahexaenoic acid (DHA). Because of their opposing molecular and metabolic actions, *n*-3 fatty acids may be able to prevent or reverse the adverse effects of fructose on hepatic lipid metabolism.

Fasting and then refeeding a high-carbohydrate diet promotes an increase in hepatic fatty acid synthase (FAS) expression and activity, hepatic TAG accumulation and secretion, and plasma TAG concentration [9]. When FO was provided in the refeeding diet, it did not prevent the increase in hepatic *de novo* fatty acid synthesis and even enhanced the hepatic TAG accumulation [10], effects that contrast with known molecular actions of *n*-3 fatty acids. Therefore, more needs to be understood about the effects of *n*-3 fatty acids in the contexts of refeeding following fasting and a background high carbohydrate diet. As such, this study aimed to clarify the effects of *n*-3 fatty acids on hepatic lipid metabolism of rats during fasting and refeeding a high-fructose diet. FO was provided in the diet before the fasting period. The effects of the FO rich diet were evaluated in the *post-prandial* (fed) period, after a 48 h long fasting period, and after refeeding on a high fructose diet following fasting. It was hypothesised that inclusion of FO in the diet would counter the adverse effects of the high fructose diet on hepatic lipid metabolism.

## 2. Experimental Section

### 2.1. Animals and Diets

**Figure 1:**
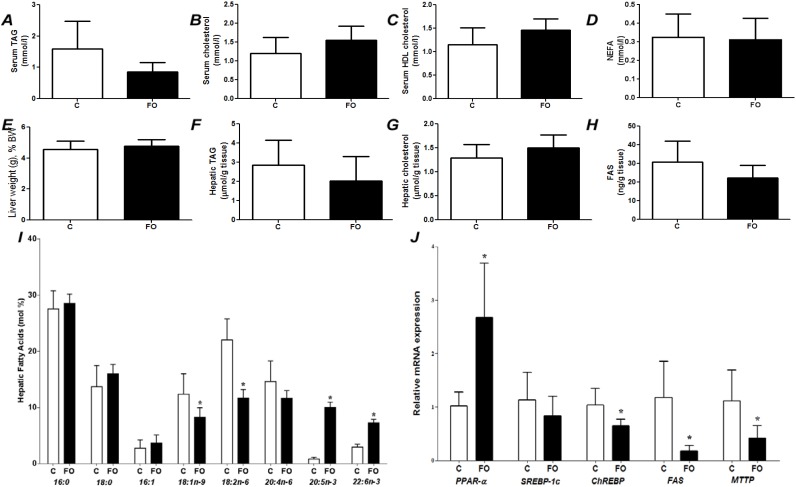
Fed state (*post-prandial*) parameters of rats fed a control (C) or fish oil (FO) diet for 7 days. (**A**) Serum triacylglycerol (TAG); (**B**) serum total cholesterol; (**C**) serum HDL cholesterol; (**D**) serum non-esterified fatty acids (NEFA); (**E**) liver weight as a percentage of body weight; (**F**) hepatic TAG; (**G**) hepatic total cholesterol; (**H**) hepatic content of fatty acid synthase; (**I**) hepatic fatty acid profile; (**J**) hepatic gene expression. PPAR-α—Peroxisome proliferator-activated receptor α; SREBP-1c—sterol regulatory element binding protein-1c; ChREBP—carbohydrate responsive element binding protein; FAS—fatty acid synthase; MTTP—microsomal triglyceride transfer protein. * Indicates significantly different from control (*p <* 0.05).

**Figure 2:**
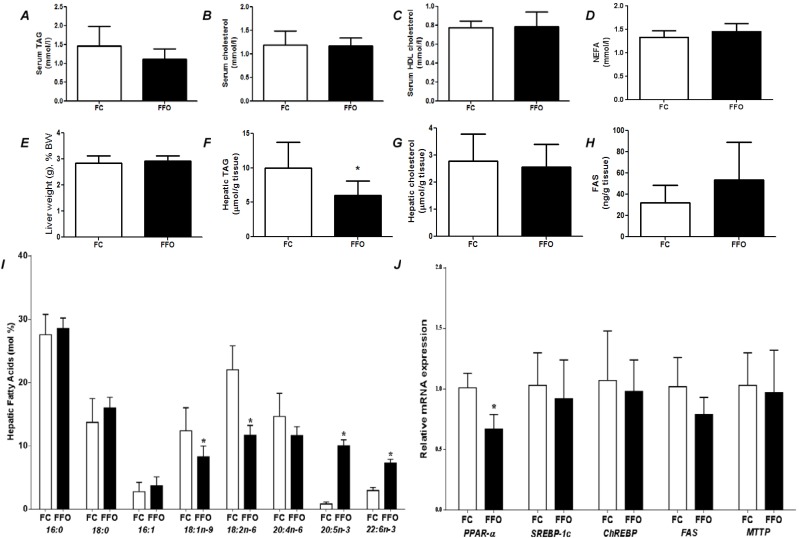
Fasting state parameters of rats fed a control (FC) or fish oil (FFO) diet for 7 days prior to a 48 h fast. (**A**) Serum triacylglycerol (TAG); (**B**) serum total cholesterol; (**C**) serum HDL cholesterol; (**D**) serum non-esterified fatty acids (NEFA); (**E**) liver weight as a percentage of body weight; (**F**) hepatic TAG; (**G**) hepatic total cholesterol; (**H**) hepatic content of fatty acid synthase; (**I**) hepatic fatty acid profile; (**J**) hepatic gene expression (see Figure 1 for abbreviations). * Indicates significantly different from control (*p <* 0.05).

**Figure 3:**
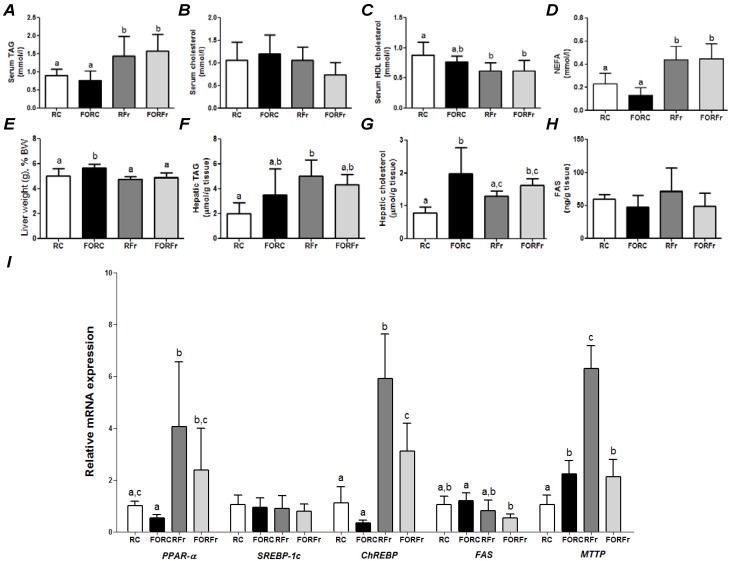
Parameters of rats refed on a control or a high-fructose diet after 7 days of feeding a control or fish oil diet and then 48 h of fasting. (**A**) serum triacylglycerol (TAG); (**B**) serum total cholesterol; (**C**) serum HDL cholesterol; (**D**) serum non-esterified fatty acids (NEFA); (**E**) liver weight as a percentage of body weight; (**F**) hepatic TAG; (**G**) hepatic total cholesterol; (**H**) hepatic content of fatty acid synthase; (**I**) hepatic gene expression (see Figure 1 for abbreviations). RC—refed control group; FORC—fish oil refed control group; RFr—refed fructose group; FORFr—fish oil refed fructose group. Bars with the same letter are not significantly different (*p <* 0.05) by Tukey’s test (^a,b,c^).

Male Wistar rats, weighing 120–150 g, were acquired from the Central Animal Care of Ribeirão Preto Campus, University of São Paulo. They were kept in a temperature and humidity controlled room and provided with food and water *ad libitum*. The light/dark cycle was also controlled and set as follows: light cycle 7 am to 7 pm; dark cycle 7 pm to 7 am. Animal handling followed the recommendations of the Brazilian College of Experiments with Animals and all procedures were approved by the Faculdade de Medicina de Ribeirão Preto Ethics Committee of Animal Experimentation on 29 March 2010, under the protocol number 013/2010. The initial experiment (Figure 1) evaluated the effects of a FO diet in rats sacrificed in the *post-prandial* period. The experiment was comprised of two groups of 8 animals each fed for 7 days on an AIN-93 diet [11]: Control (C) group or a 7% FO diet (FO group). The control diet contained 20% by weight protein (casein), 63% carbohydrate (53% starch and 10% sucrose), 7% fat (soybean oil), 5% fiber, 3.5% AIN-93G mineral mix, 1% AIN-93G vitamin mix, 0.3% L-cystine, 0.25% choline, and 0.002% tertiary-butylhydroquinone. The 7% FO diet contained FO instead of soybean oil as the lipid source. The second experiment (Figure 2) evaluated the effects of these diets following a long period (48 h) of fasting; prior to fasting, rats were fed for 7 days with the AIN-93 diet (Fasted Control (FC) group, *n =* 8) or with the FO diet (Fasted FO (FFO) group, (*n =* 8). The third experiment (Figure 3) evaluated the effects of the FO diet prior to 48 h fasting followed by refeeding on a control or a fructose-rich diet. This experiment included four groups with 8 animals each: Refed Control (RC) group (animals were fed the control diet prior to and after the fasting period); Fish Oil Refed Control (FORC) group (animals were fed the FO diet prior to fasting and refed with the control diet), Refed Fructose (RF) group (animals were fed the control diet prior to fasting and refed on a fructose-rich diet); and Fish Oil Refed Fructose (FORF) group (animals were fed the FO diet prior to fasting and were refed a fructose-rich diet). The refeeding period for all diets was 24 h and all diets were kept available to the rats until sacrifice. The fructose-rich diet followed the AIN-93 recommendations but replaced all carbohydrates (starch and sucrose) with fructose (63% by weight).

The FO used in the diets was purchased from Campestre Industry and Trade of Vegetal Oils^®^ (São Bernardo do Campo, Brazil). The fatty acid profiles of the soybean and fish oils are presented in Table 1. Vitamin, mineral mix, choline and l-cystine were purchased from Rhoster (Araçoiaba da Serra, Brazil).

All animals were sacrificed by decapitation 3 to 4 h after the beginning of the light cycle (*i.e.*, at 10 to 11 am). Blood was collected and centrifuged at 3000 rpm and 4 °C. Serum was stored at −80 °C until analysis. Livers were removed, weighed and freeze-clamped with aluminium tongs. Small pieces of liver were kept in a RNA stabilizer solution (RNAlater^®^; Qiagen, Valencia, CA, USA) until RNA extraction. To avoid exogenous RNase contamination, all materials used during the sacrifice were treated with Rnase away™ decontamination reagent (Life Technologies, Carlsbad, CA, USA).

**Table 1:** Fatty acid composition of the oils used.

Fatty Acid	Soybean Oil	Fish Oil
16:0	11.71	29.37
16:1	ND	8.4
18:0	2.89	5.94
18:1*n*-9	27.19	13.67
18:2*n*-6	5.29	16.27
18:3*n*-3	5.29	2.52
20:4*n*-6	ND	1.47
20:5*n*-3	0.37	10.13
22:6*n*-3	ND	12.24
Other	1.61	11.08

Values are means as mol percent of the total fatty acid methyl esters. ND, not detected.

### 2.2. Biochemical Analyses

TAG, total cholesterol, high-density lipoprotein (HDL) cholesterol, glucose, and aspartate aminotransferase (AST) were determined using commercial kits (Labtest Diagnóstica S.A., Vista Alegre, Brazil). Hepatic lipid extraction was made following the method of Bligh and Dyer [12]. After this, liver TAG and total cholesterol were measured by commercial kits. Serum NEFA were analysed using a commercial kit (Randox Laboratories, Crumlin, UK). Hepatic fatty acid synthase enzyme content was measured in liver homogenates with enzyme-linked immunosorbent assay (rat fatty acid synthase ELISA Kit, Blue Gene, E02F0043, Shanghai, China).

A direct transesterification method was used to determine liver fatty acid profile [13]. The fatty acid methyl esters were separated on a gas chromatograph (Shimadzu Europe, Duisburg, Germany) equipped with an AOC-20i auto-injector (Shimadzu Europe, Duisburg, Germany) using a fused silica SP-2560 column (100 m, 0.25 mm I.D., film thickness 0.20 μm). Helium was used as carrier gas and make-up gas was air. Synthetic air was used for flame ionization detection at 250 °C. Injections were made in the split mode. Fatty acid methyl ester retention times were determined by comparison with external standards (Supelco 37 component FAME Mix).

### 2.3. Hepatic Gene Expression

The hepatic gene expression was performed in duplicate in six animals per group. TRIzol (Trizol reagent-Invitrogen) was used to extract mRNA following the manufacturer’s protocol and cDNA was synthetized with a commercial kit containing random primers (High Capacity cDNA Reverse Transcription Kit, Life Technologies, Carlsbad, CA, USA). Sybr Green master mix (Power SYBR^®^ Green PCR Master Mix, Applied Biosystems, Carlsbad, CA, USA) and specific primers for each gene (Applied Biosystems, Carlsbad, CA, USA) were used to perform real-time qPCR in an ABI Prism 7500 Sequence Detection System instrument (Applied Biosystems, Carlsbad, CA, USA). 18S RNA was used as the endogenous housekeeping gene to standardized relative RNA expression. No differences between groups were observed for this gene expression. Gene expression was calculated by ΔΔC_T_ method. The sequence of the sense and antisense primers used for amplification were: PPAR-α–*F* GCAATGCACTGAACATCGAG, *R* TCTTGCAGCTTCGATCACAC; sterol regulatory element binding protein-1c (SREBP-1c)–*F* AGCACAGCAACCAGAAACTC, *R* AGGTTTCATGCCCTCCATAG; ChREBP–*F* CTTCAAAGGCCTCAAGTTGC; *R* TTCCTCCGTTGCACATACTG; FAS–*F* TCTGATCAGTGGCCTCCTTAAC, *R* CAGTGCTGAGATGTGGGAATAC; microsomal triglyceride transfer protein (MTTP)–*F* CACCGAAGTGTTTCTCGATG, *R*–CATTGACAGCCGTTATCGTG; 18S–*F* GATAAGCCCAAGCTCAATCG, *R*–TTCTGGAGTAGCGGACATTG.

### 2.4. Statistical Analysis

Unpaired *t*-test was used for comparisons between groups in Figure 1 and Figure 2. One-way analysis of variance (ANOVA) and Tukey post-test were used for data analysis for Figure 3. All statistical measurements were made with GraphPad Prism version 5.00 for Windows (GraphPad Software, San Diego, CA, USA). Differences were considered significant when *p* < 0.05. Data are expressed as mean ± standard deviation.

## 3. Results

Food intake (*p =* 0.83) and the final body weight (*p =* 0.20) were not different between C and FO groups. Figure 1 compares effects of control and FO-rich diets fed for 7 days. There were no differences between groups in liver weight, liver cholesterol, serum cholesterol, serum HDL cholesterol, or serum NEFA (Figure 1). Compared with the C group, FO tended to decrease serum TAG (*p =* 0.058; Figure 1A). Compared with the C group, FO decreased hepatic oleic and linoleic acids and increased hepatic EPA and DHA (Figure 1I). FO resulted in higher PPAR-α mRNA and lower mRNA for ChREBP, FAS and MTTP (Figure 1J).

The FC and FFO groups did not show differences in the final body weight (*p =* 0.44); body weight after experimental week (*p =* 0.49), body weight after fasting-induced weight loss (*p =* 0.32) or food intake (*p =* 0.17). Figure 2 compares effects of 48 h fasting in rats that had been fed on the control diet or a FO rich diet for 7 days prior to the fast. There were no differences between groups in liver weight, liver cholesterol, serum cholesterol, or serum NEFA (Figure 1). Compared with the C group, FO decreased liver TAG (Figure 1F) and tended to decrease serum TAG (*p =* 0.107; Figure 1A). Compared with the C group, FO resulted decreased hepatic oleic and linoleic acids and increased hepatic EPA and DHA (Figure 2I). FO resulted in lower PPAR-α mRNA and did not affect mRNA for ChREBP, FAS and MTTP (Figure 2J).

Final body weight (*p =* 0.78), body weight after experimental week (*p =* 0.78), body weight after fasting-induced body weight loss (*p =* 0.57), food intake (*p =* 0.31) and food intake after fasting (*p =* 0.22) were not different between RC, FORC, RFr and FORFr groups. Figure 3 compares the effects of 24 h refeeding on the control diet or a fructose-rich diet of rats fasted for 48 h; prior to fasting the rats had been fed on either the control diet or the FO rich diet. Compared with the control diet, refeeding on the high-fructose diet after a fasting period resulted in higher serum and hepatic TAG and serum NEFA and lower serum HDL cholesterol (Figure 3). Also, hepatic gene expression was altered by refeeding on the high fructose diet with higher expression of mRNA for PPAR-α, ChREBP and MTTP (Figure 3I). If the animals had been fed on the FO rich diet prior to the fast, there were no significant differences from the control group for serum metabolites but mRNA for ChREBP and MTTP was lower (Figure 3). For animals refed on the control diet, there was very little effect of the dietary experience prior to the fast (Figure 3). Rats that had been fed on a FO diet had higher liver EPA and DHA than those fed the control diet and this difference was seen whether the animals were refed on the control diet or the fructose-rich diet (Table 2).

**Table 2:** Hepatic fatty acid profile of rats refed on a control or a high-fructose diet after 7 days of feeding control or fish oil diet and then 48 h of fasting.

Fatty Acid	RC	FORC	RFr	FORFr
16:0	28.47 ± 2.43 ^a^	28.07 ± 2.05 ^a,b^	26.57 ± 2.38 ^a,b^	25.08 ± 1.02 ^b^
16:1	3.70 ± 0.99	4.28 ± 1.01	3.99 ± 1.83	2.75 ± 0.63
18:0	10.27 ± 1.92	10.28 ± 1.30	10.10 ± 3.21	10.40 ± 1.52
18:1*n*-9	15.19 ± 1.75	14.28 ± 1.76	16.20 ± 3.65	13.69 ± 1.92
18:2*n*-6	24.42 ± 4.14 ^a,b^	19.64 ± 1.73 ^a^	25.30 ± 3.74 ^b^	22.71 ± 1.59 ^a,b^
20:4*n*-6	11.29 ± 1.97	9.38 ± 1.45	10.51 ± 3.61	8.98 ± 1.32
20:5*n*-3	0.26 ± 0.07 ^a^	4.06 ± 1.03 ^b^	0.62 ± 0.20 ^a^	4.55 ± 1.32 ^b^
22:6*n*-3	2.74 ± 0.57 ^a^	6.50 ± 1.21 ^b^	2.58 ± 0.95 ^a^	7.92 ± 1.13 ^b^

Data are means as mol percent of the total fatty acid methyl esters. Values across a row marked with the same letter are not significantly different (*p <* 0.05) by Tukey’s test (^a,b^). RC—refed control group; FORC—fish oil refed control group; RFr—refed fructose group; FORFr—fish oil refed fructose group.

## 4. Discussion

It is well known that the long chain *n*-3 PUFAs found in FO (EPA and DHA) can affect hepatic metabolic processes, but the precise effects across different physiological situations are not fully understood. Here we examined the effect of including FO in the diet of rats on hepatic metabolic responses to feeding, to fasting for 48 h, and to refeeding on a control or high fructose diet following a period of fasting. In support of this approach, the effect of FO varied among the different situations. Marked hepatic incorporation of long chain *n*-3 fatty acids (EPA and DHA) occurred with FO feeding for 7 days. Since EPA and DHA are important substrates for synthesis of bioactive lipid mediators and can control expression of genes involved in glucose and lipid metabolism, this incorporation underpins the effects seen [14,15]. Hence, it is important that hepatic EPA and DHA levels remained elevated after a 48 h fast followed by a 7 days of refeeding period on diets not providing EPA and DHA. This means that functional effects of long chain *n*-3 PUFAs can be retained for a period after they are excluded from the diet. However, eventually levels of EPA and DHA will return to the basal state, and their functional effects will consequently be lost.

FO feeding was able to change hepatic gene expression, decreasing mRNA levels for genes such as ChREBP and FAS and increasing PPAR-α in the *post-prandial* (fed) state. Also, mRNA for MTTP, an enzyme responsible for lipoprotein assembly, was decreased in the FO group. Thus, in the normally fed state the effect of *n*-3 fatty acids is to decrease hepatic *de novo* fatty acid and TAG synthesis and assembly of TAG-rich lipoproteins and to promote fatty acid β-oxidation. Consequently, FO feeding results in lower concentrations of both hepatic and serum TAG in the fed state, although in the current study this effect of FO was not statistically significant due to the low number of animals used. Subsequent fasting of the rats reversed the effects of FO on hepatic gene expression with no differences in ChREBP or FAS mRNA compared with the control fasted group and a lower PPAR-α mRNA compared with control (fasted). Nevertheless, the effect of FO on serum and liver TAG was retained in the fasted state. FO is well known to lower fasting serum TAG concentrations in both experimental animals [16] and humans [17], acting mainly through effects on the liver [18].

Refeeding of rats on a fructose-rich diet greatly enhanced hepatic mRNA for PPAR-α, ChREBP and MTTP and serum TAG compared with refeeding on the control diet. Thus, the effect of high fructose refeeding is to promote hepatic fatty acid synthesis and TAG-rich lipoprotein assembly and so to increase TAG in the circulation. Feeding FO prior to the fasting period partly prevented the effects of the fructose feeding on ChREBP and MTTP mRNA and also resulted in lower FAS mRNA compared with what was observed with control feeding prior to the fast. Thus, the effect of prior FO feeding is to reduce hepatic *de novo* fatty acid synthesis and TAG-rich lipoprotein assembly when rats are refed on a high fructose diet.

Fasting is known to increase the hepatic expression of PPAR-α and PPAR-α target genes resulting in enhanced β-oxidation [19,20]. Although FO increased PPAR-α mRNA in the fed state it reduced PPAR-α in the fasted state, compared with the control diet. PPAR-α appears to be important in maintenance of a homeostatic metabolic response during fasting [21]. Hence the effects of prior FO feeding on the PPAR- α response to fasting seem paradoxical. However, in the present study, rats fed a FO diet prior to being fasted presented less hepatic TAG compared to rats earlier fed a control diet. They also had higher hepatic EPA and DHA compared to fasted control animals. Long chain *n*-3 fatty acids are able to modulate β-oxidation through PPAR-α independent pathways [22], which may be an important mechanism in the fasted state. A human study showed that fasting for 48 h induced β-oxidation genes and suppressed lipogenic genes in peripheral blood mononuclear cells without an upregulation of PPAR-α gene expression [23]. Thus β-oxidation can be induced without the need for PPAR-α induction, a conclusion supported by the current study.

It is well reported that fructose consumption induces SREBP-1 and ChREBP and hepatic *de novo* lipogenesis, which creates fatty acids to be assimilated in TAG [24]. In the current study (Figure 3), refeeding fasted rats on a high-fructose diet generated an increase in serum and hepatic TAG and raised hepatic ChREBP and MTTP expression.

Delzenne and collaborators showed that refeeding rats on a high-carbohydrate diet promoted an increase in liver TAG in animals previously fasted for 48 h. Although including FO in the refeeding diet lowered serum TAG, it was not able to prevent lipogenesis induction and exacerbated hepatic TAG accumulation [10]. In the present study, despite the effect of FO on hepatic gene expression, it did not prevent the fructose induced increase in serum and hepatic TAG. Thus, our findings support the earlier work [10].

*Post-prandial* (fed) periods are characterized by relatively low circulating NEFA, while fasting induces breakdown of adipose tissue TAG resulting in an increase in serum NEFA [9]. This is seen in the current study where NEFA concentrations averaged 0.3 mmol/L in the fed state (Figure 1) and between 1 and 1.5 mmol/L in the fasted state (Figure 2). Subsequent refeeding decreased NEFA concentrations (Figure 3). However, refeeding on a high fructose diet was less effective, suggesting a degree of insulin resistance at the adipose tissue level with the high fructose refeeding. This is consistent with other observations [25]. In the current study prior FO feeding did not prevent this effect of the high fructose diet.

We have considered that any effects of the FO diet are due to the presence of the biologically active *n*-3 fatty acids, EPA and DHA. However, FO also contains a high proportion of palmitic acid (16:0) which may also be biologically active. For example, palmitic acid can induce endoplasmic reticulum stress and increases synthesis of ceramides and reactive oxygen species [26]. Therefore it is important to note that, despite the high amount of palmitic acid in the FO, the hepatic incorporation of this fatty acid was not different between rats fed diets containing FO or the control diet. Therefore, effects of the FO diet seen in this study are unlikely to be due to palmitic acid and are more likely due to EPA and DHA which were well incorporated into the liver in FO groups.

## 5. Conclusions

Hepatic effects of a FO rich diet are seen in the fed state and after 48 h fasting. Furthermore, some effects of FO feeding are seen even after 7 days of refeeding on a diet not containing any FO following a 48 h fast. In the fed state, long chain *n*-3 PUFAs act to promote fatty acid β-oxidation, partitioning substrates away from *de novo* lipogenesis and assembly of TAG rich lipoproteins, consequently resulting in lower hepatic and serum TAG concentrations than seen with a comparator duet. Induction of PPAR-α seems to be important for these effects. In the fasted stated, prior feeding with FO still results in lower hepatic and serum TAG, but this seems to be a PPAR-α independent effect. Prior feeding of FO has effects that are seen after 7 days of refeeding on a high fructose diet following a 48 h fast. However, these effects do not prevent the fructose-induced elevation in hepatic and serum TAG. In conclusion, the precise effects of FO on hepatic metabolic responses depend upon the physiological state (fed, fasted, fasted and refed) and may be retained for some time without feeding a FO diet. Furthermore, long chain *n*-3 fatty acids can modify some components of the hepatic lipid metabolic response to later feeding with a high fructose diet.
